# Beyond the guidelines management of juvenile idiopathic arthritis: a case report of a girl with polyarticular disease refractory to multiple treatment options and Leri Weill syndrome

**DOI:** 10.1186/s12887-021-02494-6

**Published:** 2021-01-15

**Authors:** Vana Vukić, Ana Smajo, Mandica Vidović, Rudolf Vukojević, Miroslav Harjaček, Lovro Lamot

**Affiliations:** 1 Department of Pediatrics, University of Zagreb School of Medicine, Zagreb, Croatia; 2 Division of Clinical Immunology and Rheumatology, Department of Pediatrics, Sestre milosrdnice University Hospital Center, Zagreb, Croatia; 3 Department of Diagnostic and Interventional Radiology, Sestre milosrdnice University Hospital Center, University of Zagreb, Zagreb, Croatia

**Keywords:** Case report, Juvenile idiopathic arthritis, Leri Weill syndrome, Madelung deformity, Tofacitinib.

## Abstract

**Background:**

The last two decades brought new treatment options and high quality guidelines into the paediatric rheumatologic practice. Nevertheless, a number of patients still present a diagnostic and therapeutic challenge due to combination of vague symptoms and unresponsiveness to available treatment modalities.

**Case presentation:**

We report a case of sixteen years old girl suffering from polyarticular type of juvenile idiopathic arthritis refractory to multiple treatment options. She first presented at the age of 4 with swelling and contractures of both knees. Her symptoms were initially unresponsive to nonsteroidal anti-inflammatory drugs and progressed despite treatment with intraarticular and systemic glucocorticoids and methotrexate. Throughout the years, she received several biologics together with continuous administration of nonsteroidal anti-inflammatory drugs and disease modifying anti-rheumatic drugs as well as intraarticular and systemic glucocorticoids in disease flares. However, none of this options  provided a permanent remission, so various other modalities, as well as other possible diagnoses were constantly being considered. Eventually she became dependent on a daily dose of systemic glucocorticoids. In 2018, the treatment with Janus kinase inhibitor tofacitinib was initiated, which led to gradual amelioration of musculoskeletal symptoms, improvement of inflammatory markers and overall well-being, as well as to the weaning of systemic glucocorticoids. As the swelling of the wrists subsided for the first time in many years, Madelung’s deformity was noticed, first clinically, and later radiographically as well. Genetic analysis revealed short-stature homeobox gene deficiency and confirmed the diagnosis of Leri Weill syndrome.

**Conclusions:**

This case report emphasizes the need for reporting refractory, complicated cases from everyday clinical practice in order to build-up the overall knowledge and share experience which is complementary to available guidelines. Individual reports of difficult to treat cases, especially when additional diagnoses are involved, can be helpful for physicians treating patients with common rheumatological diseases such as juvenile idiopathic arthritis.

## Background

Joint pain and/or swelling with limited range of motion is a common manifestation of many paediatric diseases, most notably wide range of rheumatic conditions. If both of these symptoms are present for longer than 6 weeks in a patient younger than 16 years of age, a diagnosis of juvenile idiopathic arthritis (JIA), the most common childhood rheumatic disease, should be considered [1]. JIA is heterogenous disease that encompasses different subtypes of childhood arthritis defined depending on the number of affected joints and/or presence of the enthesitis and/or sacroiliitis. Nevertheless, alongside JIA, there is a wide range of loosely related noninflammatory causes of a swollen joint in children, especially in the absence of clinical signs of inflammation. Lysosomal storage diseases (LSD) such as mucopolysaccharidosis type I (MPS I), Gaucher disease type I and Fabry disease all have prominent musculoskeletal symptoms early in the course of the disease, and are often first seen by a pediatric rheumatologist [2–8]. However, the underlying mechanism of those disorders does not directly involve the immune mediated inflammatory response, but rather an inflammation caused by genetic defects and subsequent perturbations at the protein level. More specifically, in Gaucher disease, bone marrow infiltration with histiocytes causes acute attacks of pain, which may be mistaken for arthritis in the vicinity of a joint [5, 8]. In Fabry disease, episodes of neuropathic pain in hands, feet, wrists, ankles (acroparesthesias), often associated with fever, malaise and elevated inflammation markers, can mimic a rheumatic condition, such as an inflammatory arthritis [3, 9]. Finally, joint stiffness and contractures are characteristic for some types of MPS, the so called „attenuated“ forms like Hurler-Scheie syndrome, which have a less severe presentation and progress silently over the years, making the diagnosis a challenge [3, 10, 11]. On the other hand, some congenital conditions, such as Madelung and Madelung-type deformities resulting from the premature closure of the medial volar aspect of the distal radial physis, might cause similar symptoms [12]. Hence, it is not a rarity that some of the children with LSD and congenital deformities are treated for prolonged periods of time as having an inflammatory arthritis, despite the lack of appropriate response [13].

With regard to the treatment of JIA, various modalities emerged over the last two decades, revolutionizing the pediatric rheumatology practice [14]. Majority of the presently available guidelines recommend a step-up approach, starting with nonsteroidal anti-inflammatory drugs (NSAID) and intraarticular glucocorticoid injections (IAGI), followed by conventional and biologic disease modifying anti-rheumatic drugs (cDMARD and bDMARD), respectively [15]. Moreover, despite many known adverse effects, systemic glucocorticoids (GC) are still being used as an important therapeutic option for wide range of complications associated with JIA (e.g., macrophage activation syndrome, myocarditis, pericarditis, pleuritis, peritonitis, uveitis and severe anaemia), as well as a bridge therapy in severe forms of JIA before the full effect of other treatment modalities has been achieved [16]. Those modalities nowadays primarily involve tumor necrosis factor alpha (TNFα) inhibitors (TNFi), such as etanercept, adalimumab and infliximab, and non-TNFi, such as anti-interleukin-6 (anti-IL-6) agent tocilizumab and a selective T-cell co-stimulation modulator abatacept [17, 18]. Furthermore, new medications are continuously being investigated [19, 20].

Together with the expansion of new treatment modalities, efforts have been made to introduce the treat-to-target (T2T) model in pediatric rheumatologic practice [21]. Signs and symptoms control, prevention of structural damage of joints and optimization of linear growth and pubertal development, as well as abolition of inflammation, have all been set as treatment goals. Essentially, this model advocates that therapy should be revised and adjusted based on regular disease activity assessments to reach and maintain the treatment target. Special attention should be paid to preventing or minimizing the side effects of systemic GC given their negative effect on growth and pubertal development. Consequently, their long-term use to maintain treatment target should be avoided, especially considering that GC dependence demonstrates the inadequacy of chosen treatment. The shared decision making, as well as multidisciplinary approach, have been recognized as exceptionally valuable for assurance of better adherence to treatment and subsequently improvement of outcome and overall prognosis.

Unfortunately, despite all these achievements, only 14 % of patients with rheumatoid factor (RF) negative and 0 % with RF positive polyarticular JIA achieves the remission off medications within five years, implying that JIA treatment requires a long-lasting commitment [22]. Hence, it comes as no surprise that every clinician involved in the care of children with JIA patients eventually sees one with disease not responding to acclaimed treatment options (i.e. NSAIDs, cDMARDS, bDMARDS) [15]. At that point, the consideration of additional treatment modalities seems like a valid course of action, but sometimes alternative diagnosis should be considered as well. Here, we present one such case, a girl with long standing polyarticular JIA refractory to many standard treatment modalities, with symptoms suggestive of other diseases.

## Case presentation

A four-year-old first came to our attention in February 2008 due to painful swelling and contractures of both knees. Her symptoms started a year earlier and were not associated with any discernible trigger such as infection or trauma. Moreover, the symptoms were not responding to NSAIDs and she soon developed a severe morning stiffness lasting for up to three hours. Her birth history as well as psychomotor development prior to disease evolution was unremarkable. She was born as a first child into a family of non-consanguine parents, with no relatives having the similar symptoms.

The initial laboratory findings showed persistently elevated inflammatory markers (erythrocyte sedimentation rate (ESR) up to 100 mm/h and C-reactive protein (CRP) up to 100 mg/dL), with negative RF and antinuclear antibody (ANA) screen, and normal immunoglobulin levels. Despite the initial treatment with GC and methotrexate (MTX), her symptoms progressed affecting elbows, wrists, ankles and small joints of both hands. Moreover, she developed a severe uveitis of the left eye. In November 2008 biologic therapy with infliximab was started, with initially good response. Unfortunately, this lasted only for a few months and frequent relapses necessitated switching to adalimumab in April 2011. Again, there was an initial period of remission followed by progressive exacerbation characterized by swelling and pain in some joints, and persistent contracture of others. Other diagnosis, such as mucopolysaccharidosis and systemic lupus erythematosus (SLE) were suspected, but metabolic and immunological screening were negative, respectively. Beside ESR and CRP, the increased values of IL-6 (up to 75 pg/mL) and TNF-alfa (up to 20 pg/mL) were measured.

**Fig. 1:**
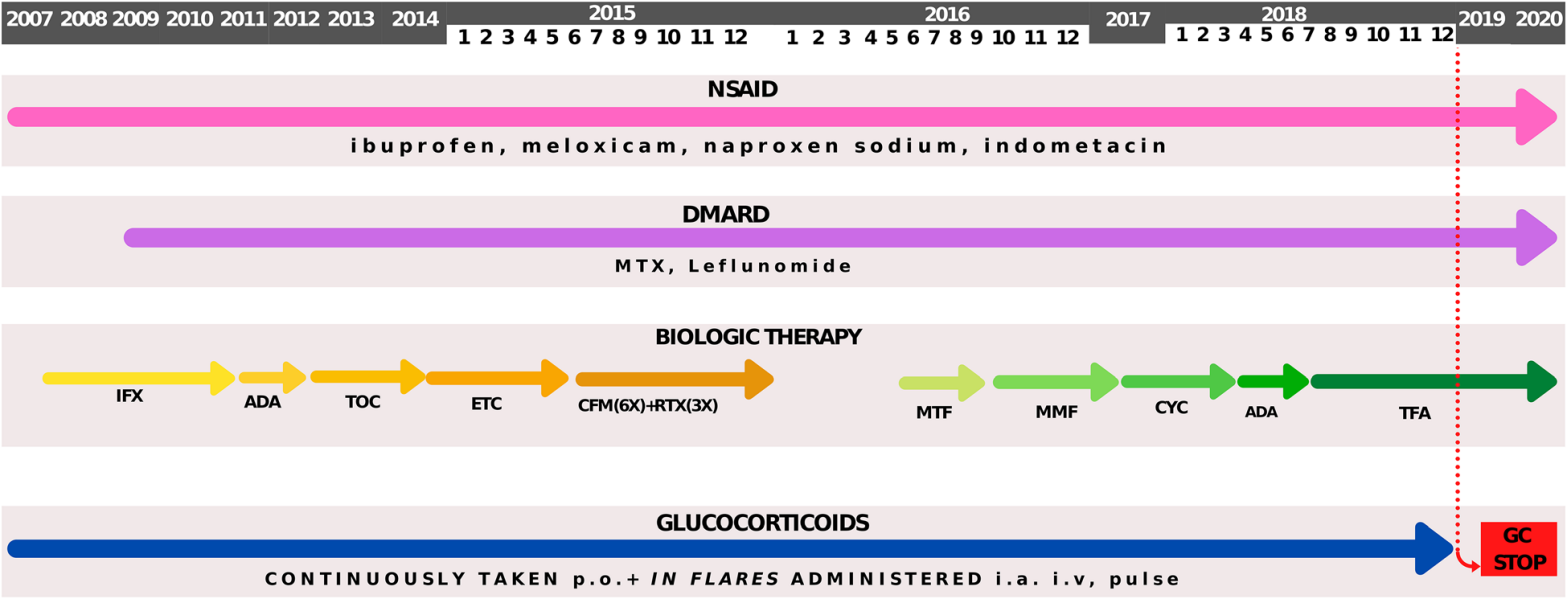
Schematic representation of treatment modalities during the time. 2007 – 2020 - the period of treatment, NSAID - nonsteroidal anti-inflammatory drug, IFX - infliximab, ADA - adalimumab, TOC - tocilizumab, ETC - etanercept, DMARD - disease modifying anti-rheumatic drug, MTX - methotrexate, CFM - cyclophosphamide, RTX - rituximab, MTF - metformin, MMF - mycophenolate mofetil, CYC - cyclosporine, TFA - tofacitinib, p.o. - per os, i.a. - intraarticular, i.v. - intravenous, GC - glucocorticoids

In November 2012 therapy was cycled to non-TNFi, tocilizumab, which led only to a short period of remission. Finally, in September 2014, etanercept was introduced, again with the lack of permanent response. Along with four different bDMARDs she continuously received cDMARD methotrexate, and for a short period of time leflunomide. During the periods of disease flare, bridge therapy with intraarticular and/or systemic GC was used, and soon she was dependent on a daily dose of GC. Eventually, this led to the development of iatrogenic Cushing syndrome with characteristic appearance, growth retardation and low bone mineral density, regardless of the vitamin D and ibandronic acid therapy. Since every attempt to wean off GC inevitably led to disease flare, from June to December 2016 she received cyclophosphamide (6x) and rituximab (3x), again without achieving a sustained remission. Afterwards, for a short period of time she was given metformin but without an appropriate improvement in musculoskeletal symptoms (Fig. 1).

**Fig. 2:**
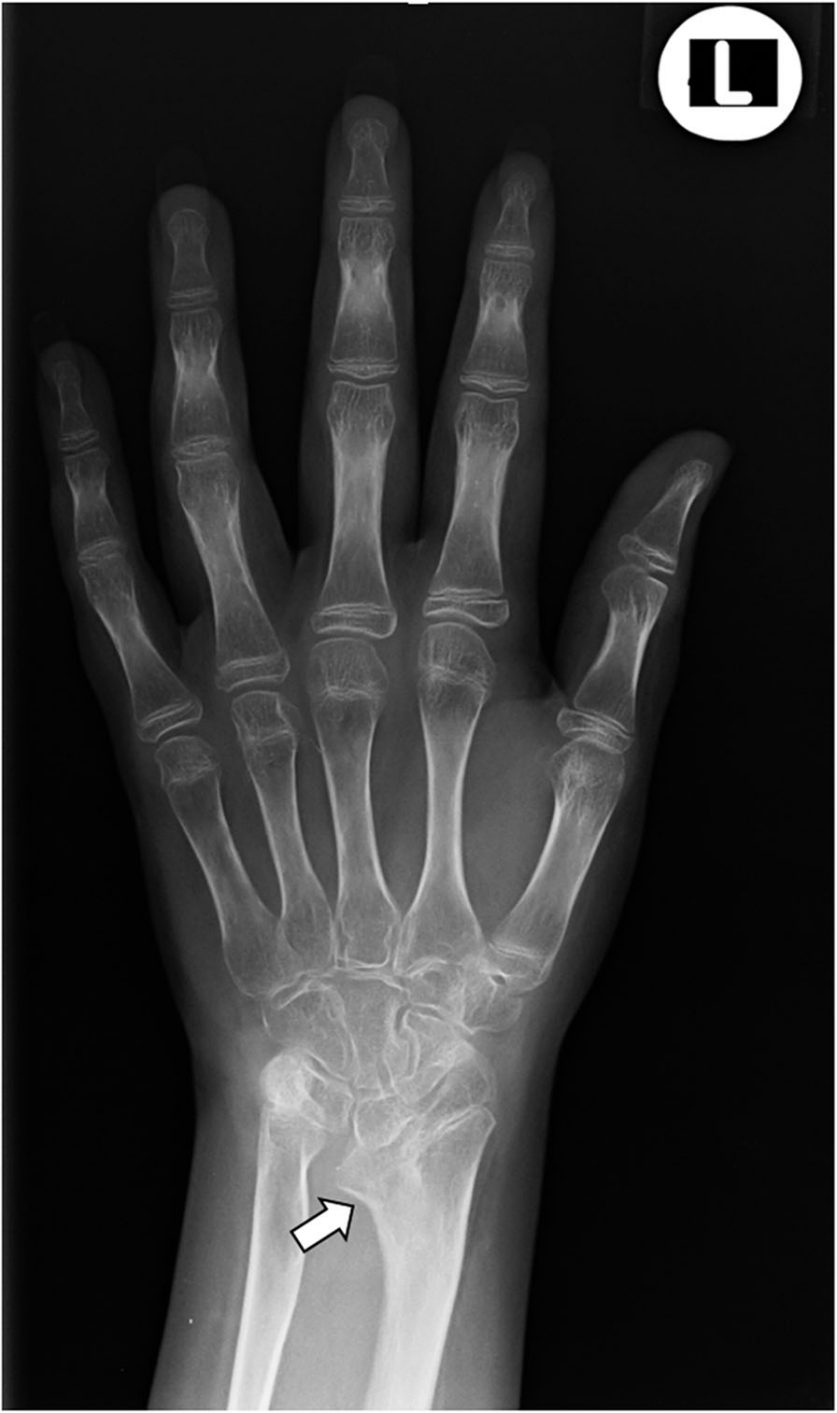
The anteroposterior radiograph of left hand at the age of 14. Note the increased volar angulation of distal radius, wedge shaped carpus with proximally positioned lunate and a characteristic notch on the distal radius (white arrow), which are the features of the Madelung deformity [12]

**Fig. 3:**
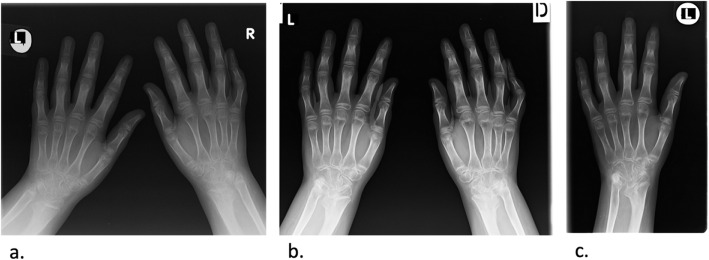
The anteroposterior radiograph of both hands at the age of 10 (**a**) and 13 (**b**), and of the left hand at the age of 14 (**c**). Note the bowing of the distal radius, an increased radial inclination (~ 30°) with the deformation of the carpus that acquired a triangular appearance and widening of the distal radial-ulnar joint bilaterally, which are the typical features of Madelung deformity. Dorsal subluxation of the ulnar head is not seen as lateral images of the wrist were not taken. Osteopenia of carpal bones and periarticular osteopenia of MCP, PIP and DIP joints related to JIA are present. No relevant changes are observed during the time

**Fig. 4:**
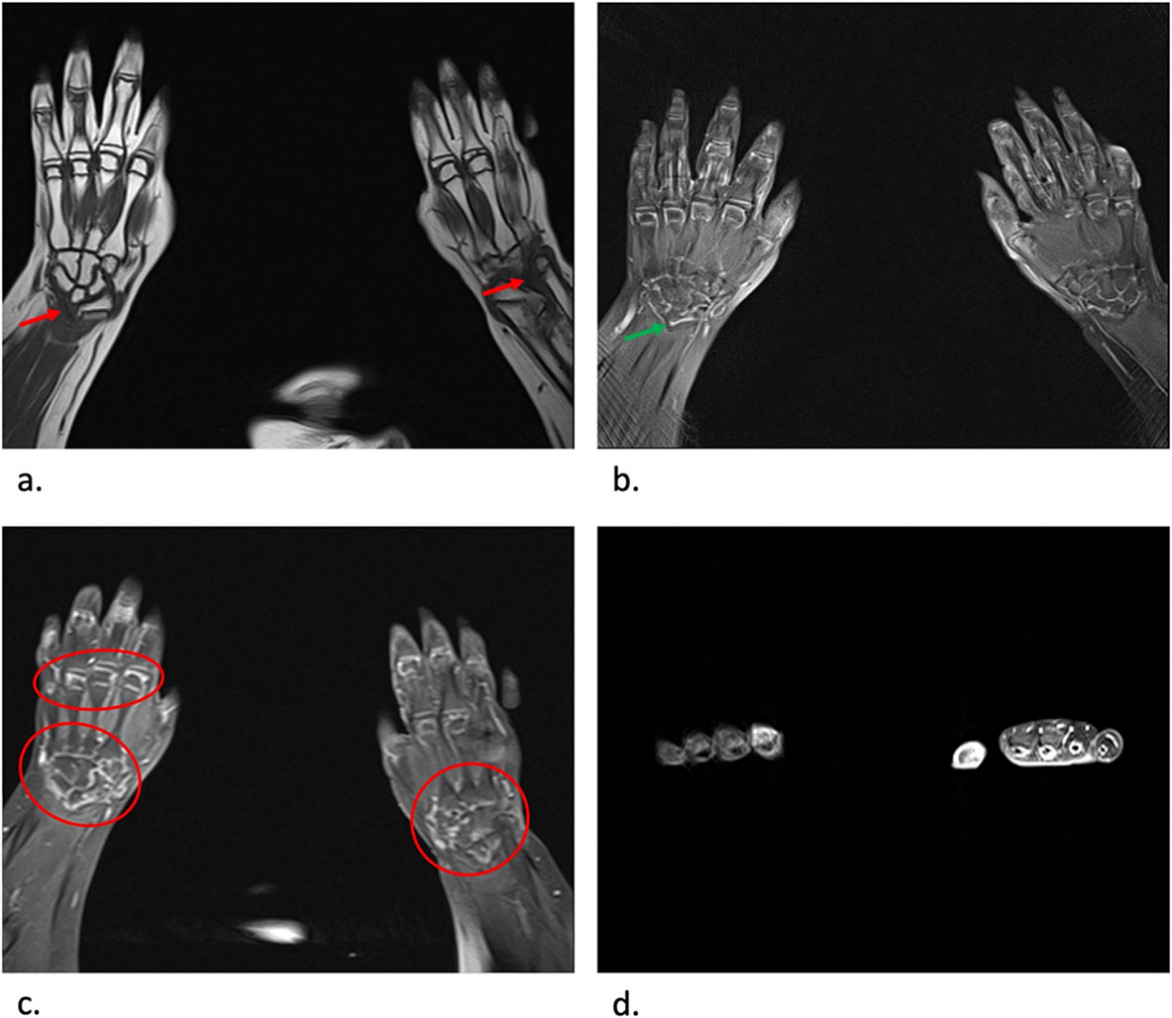
MRI coronal T1-weighted (**a**), proton density BLADE fluid sensitive sequence (**b**), post contrast T1-weighted coronal fat sat sequence (**c**) and post contrast T1-weighted axial fat sat sequence (**d**) images of both hands at the age of 13. Note the radiotriquetral ligament (red arrow) and Vickers ligament (green arrow) (**a**, **b**). Note the inflammatory changes characterized by postcontrast imbibition in carpal joints and MCP joints (red circle), as well as tenosynovitis of flexor tendons related to JIA, more prominent on the left hand (**c**, **d**)

During the 2017, at the age of 13, the trial of mycophenolate mofetil (MMF) followed by the trial of cyclosporin was initiated, but the patient nevertheless remained GC dependent. Both shoulders, elbows, radiocarpal joints, metacarpophalangeal (MCP), proximal interphalangeal (PIP), distal interphalangeal (DIP) joints and both knees had restricted range of movement, and repeated IAGI were necessary to alleviate the symptoms. Finally, in 2018, the treatment with Janus kinase (JAK) inhibitor tofacitinib was initiated, which lead to gradual amelioration of musculoskeletal symptoms and improvement of inflammatory markers and overall well-being, as well as to the weaning of systemic GC. Moreover, as the swelling of the wrists subsided for the first time in many years, Madelung deformity was noticed by clinical examination. Interestingly, it was only then, in 2019, that the deformity was for the first time described on x-ray (Fig. 2), although x-ray and MRI imaging of both hands were previously performed on many occasions in order to assess the inflammation (Figs. 3 and 4). Nevertheless, subsequent analysis by experienced musculoskeletal radiologist revealed characteristic bilateral signs of Madelung deformity dating back in 2015 and 2017 (Fig. 3), with the MRI showing the Vickers and radiotriquetral ligament (Fig. 4). The finding of those two ligaments allowed the distinguishing between Madelung deformity and pseudo-Madelung deformity, which includes post-traumatic and post-infective forms, forms associated with Turner syndrome, multiple hereditary exostoses and Ollier disease [23]. Unfortunately, due to the parent’s refusal, no further radiological assessment was performed, so we have not documented the other important aspects of Madelung deformity, such as radial shortening and diaphysis bowing, nor the mesolimbic shortening of limbs characteristic for Leri Weill syndrome.

Although our patient was simultaneously followed by pediatric endocrinologist from age of 11, her short stature, along with delayed menarche, and Cushingoid appearance, was attributed to the prolonged use of GC. It was only after the Madelung’s deformity was observed that genetic causes, primarily Leri Weill syndrome, were taken into consideration. Genetic analysis was performed by commercially available SALSA MLPA Probemix P018 SHOX *(MRC-Holland, Amsterdam, The Netherland)* according to the manufacturer’s recommendations. The MLPA mix included probes for each exon of SHOX, one probe just before the promoter region as well as probes covering a region downstream of the gene. The results revealed one copy of sixteen probes (10 probes for Xp22-PAR1 from CNE2 to CNE9, 4 probes for SHOX area downstream, 1 probe for CRLF2 in PAR1 region and 1 probe for CSF2RA in PAR1 region), with the size of the smallest deletion of 766,5 kb. Based on the genetic testing and imaging findings of Madelung deformity, the diagnosis of Leri Weill syndrome was established, and parents were advised to undergo further genetic testing of both the patient and themselves, which they rejected.

Currently, our patient has many consequences of the adverse course of the disease and prolonged GC treatment, such as joint contractures of elbows, wrists, DIPs, PIPs, hips, knees and MCPs and low bone density, respectively. She developed secondary sex characteristics only after the therapy with estradiol was initiated at the age of 14. Nevertheless, after the discontinuation of GC and subsequent discontinuation of estradiol, she finally had a menarche at the age of 15, along with a long-awaited growth spurt.

## Discussion and Conclusion

Children with rheumatic diseases, their families, as well as their treating physicians are dealt with numerous issues and dilemmas regarding either the disease itself or ongoing treatment modalities. Besides, the diagnosis of rheumatic diseases in children is regularly made by excluding wide range of other diseases, with no pathognomonic tests and/or criteria. Therefore, even after the classification criteria are fulfilled, the diagnosis should be revised if new symptoms emerge or if the recommended treatment options are failing.

In our patient, a variety of steroid-sparing agents with different mechanisms of action have been employed with limited or no clinical success. Some of these agents were used in line with current treatment recommendations, but many were used based on anecdotal reports [24–28]. Finally, due to signs of systemic inflammation characterized by increased inflammatory markers (CRP and ESR) and cytokines (IL-6 and TNF-alfa), which is indicative for the activation of JAK/STAT pathway, treatment with tofacitinib, a first generation JAK inhibitor, was initiated with a good clinical response. Several clinical trials in adults with rheumatoid and psoriatic arthritis have given solid evidence about the use of tofacitinib, while the results of a phase 3 randomized double blind placebo controlled withdrawal study in patients with polyarticular JIA showed improvement in symptoms, less disease flares and improved functional ability, together with a clinical amelioration of disease activity [29, 30]. Moreover, tofacitinib is an oral agent, and the challenge of using biologics requiring injection or infusion for an extended length of time, especially in children, should not be overlooked.

Along with the various treatment modalities, the different diagnosis was constantly being considered in our patient. Firstly, due to persistent contracture in some joints with little or no signs of swelling, LSDs such as mucopolysaccharidosis type I, Gaucher disease type I and Fabry disease were investigated. Besides, other inflammatory causes like systemic lupus erythematosus were also excluded. Lastly, the diagnosis of Leri Weill syndrome characterized by deletions in SHOX gene and Madelung deformity was established. This painful deformity of the wrist was first described in 1878 by the German surgeon Otto Madelung in adolescents between the ages 8 and 14 [12]. Although initially asymptomatic, the patients often went on to develop pain, loss of grip strength and reduced mobility, which were the symptoms present in our patient even after the inflammation was tackled with tofacitinib. Moreover, the features of Leri Weill syndrome include the short stature, which was also one of the dominant finding in the presented patient. Yet, the growth deficit caused by SHOX haploinsufficiency in Leri Weill syndrome is around 2 standard deviation scores (SDS) [31], while our patient had a SDS of -5,5. Besides, due to a prolonged use of GC, our patient had a full blown Cushingoid appearance, low bone density, delayed puberty and growth retardation. Therefore, the possible explanation for the short stature and growth delay in our patient includes multifactorial aetiology. Firstly, it is well known that extended GC treatment leads to a defect in bone turnover (and formation) due to impaired osteoblastogenesis and osteoclastogenesis, and may have direct effects on the growth plate [32]. Additionally, higher prepubertal glucocorticoid level appears to delay early and late pubertal timing of healthy girls, particularly the onset of pubertal growth spurt and menarche [33]. Moreover, it has been shown that girls with polyarticular juvenile idiopathic arthritis are significantly more likely to present with short stature even 6 months after stopping the steroid therapy [34]. Finally, the product of SHOX gene is implicated in bone development and regulation of chondrocyte differentiation, which clarifies the association of SHOX gene haploinsufficiency with idiopathic short stature, as well as short stature in Turner syndrome and Leri Weill dyschondrosteosis [31].

In the presented case, the diagnosis of the Madelung deformity and Leri Weill syndrome was delayed due to the concomitant active inflammation caused by JIA taking the focus from other possible causes of pain in the wrists. However, as subsequent analysis by experienced musculoskeletal radiologist has shown, the characteristic signs of the Madelung deformity were present few years before the final diagnosis was reached, emphasizing once again the importance of multidisciplinary approach and close collaboration of many subspecialists in the care of children with rheumatic diseases. Nevertheless, this lag probably did not influence the therapeutic management in our particular patient; although positive effect on final height was observed with growth hormone therapy in patients with Leri Weill syndrome, due to the matching influence of GC and lack of agreement with her parents, this treatment option was avoided in our patient [35].

In conclusion, this case report emphasizes the difficulties and challenges in management of patient with long-standing polyarticular JIA refractory to wide range of treatment modalities. Although many high-quality guidelines are available for treatment of JIA patients, there is still need for individual reports of difficult to treat cases, especially when additional diagnosis are involved. While Leri Weill syndrome is extensively reported in the literature, to the best of our knowledge, our case report describes it for the first time along with JIA. Taking all these into account, we strongly encourage the aggregation of similar patients and establishment of the common ground that will help clinician to decide upon the introduction of treatment options outside of the contemporary guidelines.

## Data Availability

Data sharing is not applicable to this article as no datasets were generated or analysed during the current study. / Not applicable.

## Abbreviations

JIA: Juvenile idiopathic arthritis
LSD: Lysosomal storage disease
MPS: Mucopolysaccharidosis
NSAID: Nonsteroidal anti-inflammatory drug
cDMARD: Conventional disease modifying anti-rheumatic drug
bDMARD: Biologic disease modifying anti-rheumatic drug
IAGI: Intraarticular glucocorticoid injection
GC: Glucocorticoids
MAS: Macrophage activation syndrome
TNFi: Tumor necrosis factor inhibitor
T2T: Treat-to-target
RF: Rheumatoid factor
ESR: Erythrocyte sedimentation rate
CRP: C-reactive protein
ANA: Antinuclear antibody
MTX: Methotrexate
SLE: Systemic lupus erythematosus
TNFα: Tumor necrosis factor alpha
anti-IL-6: Anti-interleukin-6
MMF: Mycophenolate mofetil
MCP: Metacarpophalangeal
PIP: Proximal interphalangeal
DIP: Distal interphalangeal
JAK: Janus kinase
MRI: Magnetic resonance imaging
SHOX: Short-stature homeobox
JAK/STAT: Janus kinase/signal transducer and activator of transcription proteins
SDS: Standard deviation score
GnRH: Gonadotropin-releasing hormone
HPG: Hypothalamic-pituitary-gonadal
IFX: Infliximab
ADA: Adalimumab
TOC: Tocilizumab
ETC: Etanercept
CFM: Cyclophosphamide
RTX: Rituximab
MTF: Metformin
CYC: Cyclosporine
TFA: Tofacitinib
